# Computed tomography−based prediction of commissural positions facilitates valve-sparing aortic root replacement

**DOI:** 10.1016/j.xjtc.2025.11.007

**Published:** 2025-11-27

**Authors:** Haruo Yamauchi, Masahiko Ando, Kenji Ino, Hiroyuki Tsukihara, Gakuto Aoyama, Ichiro Sakuma, Minoru Ono

**Affiliations:** a Department of Cardiovascular Surgery, Graduate School of Medicine, University of Tokyo, Tokyo, Japan; b Radiology Center, University of Tokyo Hospital, Tokyo, Japan; c Medical Device Development and Regulation Research Center, Graduate School of Engineering, University of Tokyo, Tokyo, Japan; d Research and Development Center, Canon Medical Systems Corporation, Otawara, Japan; e Research Institute for Science and Technology, Center for Research and Collaboration, Tokyo Denki University, Tokyo, Japan

**Keywords:** commissural position, computed tomography, David reimplantation, deep-learning-based algorithm, transesophageal echocardiography, virtual basal ring

## Abstract

**Objectives:**

This study aimed to compare aortic root reimplantation planning using computed tomography (CT) with conventional methods and to assess the accuracy of automated CT measurements using a deep-learning algorithm.

**Methods:**

Twenty patients underwent David reimplantation at our hospital with CT-based planning to determine graft sizes from virtual basal ring dimensions and predict commissural positions using electrocardiogram-gated CT. In the controls (n = 20), preoperative transesophageal echocardiography determined virtual basal ring sizing, whereas surgeons intraoperatively assessed commissural positions. We also analyzed correlations between CT measurements obtained manually by experts and automatically by our deep-learning-based algorithm using 50 cases indicated for David reimplantation.

**Results:**

The CT group had a shorter aortic crossclamp time (*P* = .001). Horizontal shifting of commissures (80% vs 40%) and uneven commissural heights (95% vs 60%) were more frequently observed in the CT group than in the controls (*P* = .010) during David reimplantation. The CT group had fewer patients (25% vs 85%) and commissures (15% vs 63%) requiring intraoperative commissural position adjustments than the controls (*P* < .001). Cusp repair was required in 1 patient in the CT group and 7 patients in the control group (*P* = .018). Algorithm-based measurements showed excellent agreement with expert measurements for virtual basal ring diameters, intercommissural distances, and commissural heights (differences: −0.3 to 0.1 mm; intraclass correlation coefficients: 0.87-0.97).

**Conclusions:**

For David reimplantation, CT-based planning of graft sizes and commissural positions may reduce the need for intraoperative commissural position adjustments and cusp repair. Our deep learning−based algorithm can replace expert measurements in standardized surgical planning.

## Graphical abstract

Pictorial summary of the methods, results, and implications of this study.

**Figure:**
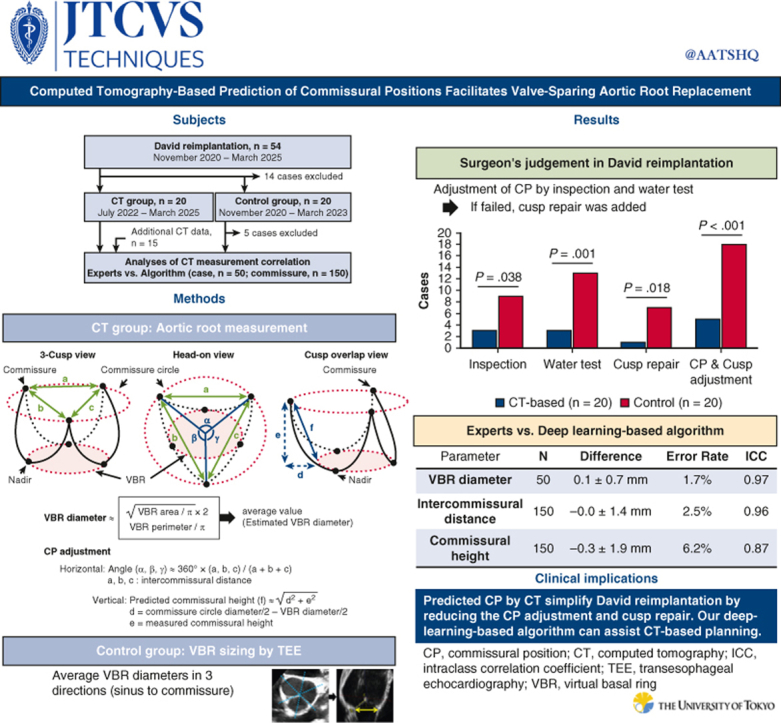

**Figure:**
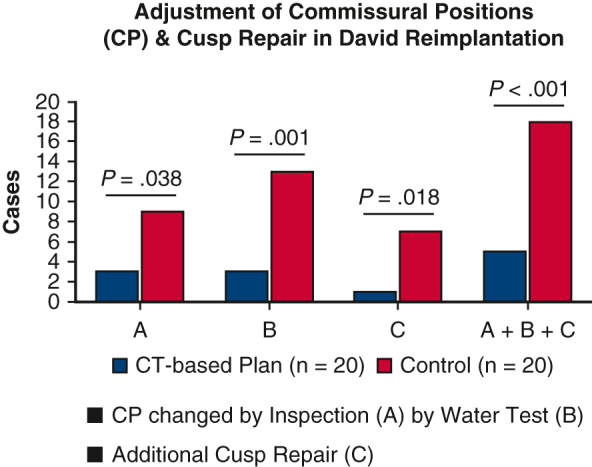
Predicting commissural positions by computed tomography facilitates David reimplantation.

**Central Message**

Predicted commissural positions (CP) by computed tomography (CT) simplify David reimplantation by reducing the CP adjustment and cusp repair. Our deep learning−based algorithm helps CT-based planning.

**Perspective**

Computed tomography (CT)-based prediction of virtual basal ring sizes and commissural positions (CP) facilitates David reimplantation more effectively than conventional methods. The CT-based planning reduced the need for CP adjustment and additional cusp repair, leading to shortened cardiac arrest times. Our deep learning−based algorithm demonstrated excellent correlations with expert measurements.

Valve-sparing root replacement (VSRR) is becoming the standard option for aortic root dilatation, with evidence of favorable long-term outcomes in high-volume centers.^1^^,^^2^ Successful VSRR requires a thorough understanding of root anatomy, including the virtual basal ring (VBR), the sinus of Valsalva (SOV), and the sinotubular junction (STJ).

High-resolution computed tomography (CT) has been used for aortic root assessment^3^, ^4^, ^5^, ^6^ and revealed disproportionate normal aortic valves with larger noncoronary sinuses and greater commissural heights (CHs) between the right and noncoronary sinuses than the others. The dimensions of aortic cusps, such as the geometric height (gH), correlate with the root sizes.^4^ Restoration of the VBR and commissural positions (CPs) that preserve the intercusp dimensional relationship is a complex but necessary process for VSRR. If the effective heights (eHs) of the cusps are insufficient as a result of prolapse, cusp repairs are necessary. However, a recent study reported that additional cusp repair adversely impacts the recurrent aortic regurgitation (AR) after VSRR.^7^ Therefore, durable VSRR depends on the experience of the surgeon and the institution.^2^^,^^8^

Standardized operative techniques with a CT-based objective aortic valve assessment are beneficial, although few studies have reported on the clinical outcomes of this approach.^6^ One of the most technically demanding elements of VSRR is the intraoperative determination of CPs. Our group recently started using electrocardiogram-gated CT images for measuring VBR and CPs in VSRR (David reimplantation) planning. To facilitate this, we developed a deep learning−based algorithm for fully automated aortic valve and root measurement.^9^^,^^10^

This study had a dual purpose. First, we aimed to compare patient outcomes after CT-based planning of David reimplantation with those of controls after conventional strategies, which included preoperative transesophageal echocardiographic (TEE) measurement of VBR and the surgeon's intraoperative judgment to determine CPs. Second, we aimed to compare our algorithm's automated CT measurements with the experts' manual measurements for exploring the potential role of artificial intelligence in surgical planning.

## Methods

### Data Collection

**Figure 1:**
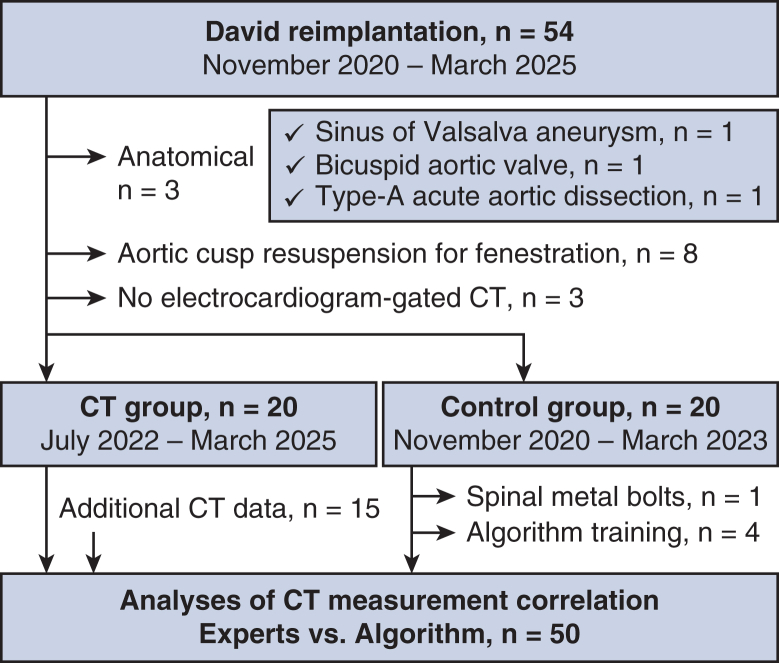
Study eligibility criteria. *CT*, Computed tomography.

This was a retrospective observational study. Figure 1 shows the participant eligibility criteria. In total, 54 consecutive patients who underwent David reimplantation at our institution between November 2020 and March 2025 were reviewed. Of these, 14 were excluded as the result of anatomical variations (n = 3), aortic cusp fenestration necessitating preventive leaflet resuspension (n = 8), or no available electrocardiogram-gated CT data (n = 3). Twenty patients underwent David reimplantation between July 2022 and March 2025 after CT-based planning. The remaining 20 patients formed the control group. One patient diagnosed with chronic type A dissection was included in each group (Table 1). This study was conducted in accordance with the tenets of the 1964 Declaration of Helsinki and its later revisions. It was approved by the Research Ethics Committee of the Faculty of Medicine, The University of Tokyo, on January 27, 2017 (approval no. 11330). The requirement for written informed consent from participants was waived by the institutional review board because patient data were retrospectively collected and anonymized, and de-identified before use.

**Table 1:** Demographics and preoperative echocardiography data

Demographics	CT group	Control group	*P* value
	(n = 20)	(n = 20)	
Age, y	39.1 ± 16.0	28.4 ± 10.7	.019∗
male, n (%)	16 (80%)	12 (60%)	.17
Body surface area, m2	1.8 ± 0.2	1.8 ± 0.3	.72
Connective tissue disease, n (%)	18 (90%)	17 (85%)	.63
History of cardiac surgery, n (%)	1 (5%)	2 (10%)	.55
Mitral valve repair	1	2	
Indication for root replacement, n (%)			1.00
Annuloaortic ectasia	19 (95%)	19 (95%)	
Chronic type A aortic dissection	1 (5%)	1 (5%)	
**TTE**	**(n = 20)**	**(n = 20)**	
LV dimension in diastole, mm	50.5 ± 6.5	50.7 ± 5.3	.94
Ejection fraction, %	61.8 ± 6.8	64.3 ± 8.3	.31
Aortic valve peak velocity, m/s	1.1 ± 0.3	1.0 ± 0.2	.81
Aortic regurgitation			.28
No-trivial, n (%)	11 (55%)	13 (65%)	
Mild, n (%)	5 (25%)	1 (5%)	
Moderate, n (%)	3 (15%)	3 (15%)	
Severe, n (%)	1 (5%)	3 (15%)	
VBR diameter, mm	24.6 ± 3.3	25.9 ± 3.5	.28
SOV diameter, mm	48.6 ± 8.4	42.9 ± 3.8	.010∗
**TEE**	**(n = 14)**	**(n = 20)**	
VBR diameter, mm	28.2 ± 3.5	28.3 ± 3.9	.92
SOV diameter, mm			
Left coronary sinus	44.9 ± 6.1	43.9 ± 6.4	.66
Right coronary sinus	46.5 ± 6.2	43.1 ± 5.7	.12
Noncoronary sinus	45.1 ± 5.7	44.7 ± 6.3	.86

Data are shown as mean ± standard deviation. *CT*, Computed tomography; *TTE*, transthoracic echocardiography; *LV*, left ventricle; *VBR*, virtual basal ring; *SOV*, sinus of valsalva; *TEE*, transesophageal echocardiography.

∗ *P* < .05 was considered statistically significant.

### Echocardiographic Evaluation

**Figure 2:**
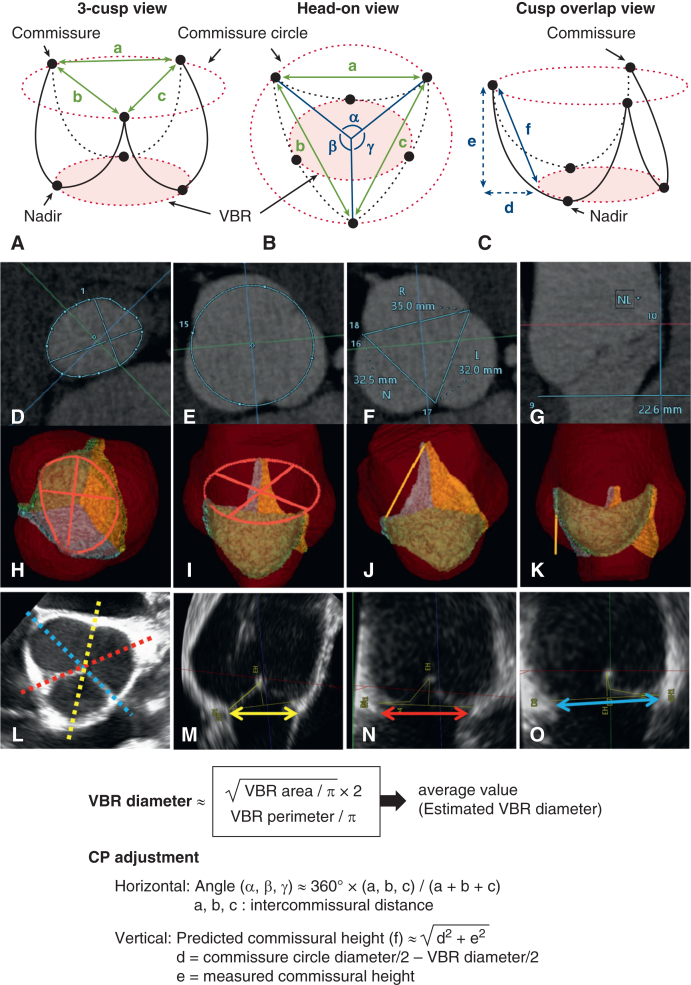
Preoperative aortic root/valve measurement parameters. Schematic drawings of the aortic root structures are shown as a 3-cusp view (A); a head-on view (B); and a cusp overlap view (C). VBR areas are depicted in *pink*. The ICDs are shown as *bidirectional green solid lines* (a-c). Angles α, β, and γ were approximated from the ICDs a-c. The predicted CH (f) (*bidirectional blue solid arrows*) was calculated from dimensions d and e (*bidirectional blue dotted arrows*) using the Pythagorean theorem. For the CT analyses, the aortic root structure is visualized in 2-dimensional images with measurement parameters (D-G) and 3-dimensional images by the algorithm (H-K), showing the VBR (D and H), CCA (E and I), ICD (F and J), and CH (G and K) in *blue*, *red*, and *orange lines*. For the TEE analysis, the VBR diameters in 3 directions between their nadirs and the opposite commissure sides are shown as bidirectional *yellow*, *red*, and *blue arrows* (L-O). The formulas used to calculate each parameter are shown in the lower panel. *VBR*, Virtual basal ring; *ICD*, intercommissural distances; *CH*, commissural heights; *CT*, computed tomography; *CCA*, commissure circle area; *TEE*, transesophageal echocardiography.

Transthoracic echocardiography (TTE) was evaluated for general cardiac functions. David reimplantation was considered if the aortic cusps had minimal deformity and the SOV exceeded 40 to 45 mm in patients with connective tissue diseases and 55 mm in those without, according to Japan Circulation Society guideline issued in 2020.^11^ Preoperative TEE was evaluated if tolerated by the patient, and multiplanar resection images of the aortic root in the end-diastolic phase were obtained from volume datasets and the average VBR diameters were measured from the nadirs of the left, right, and noncoronary cusps, to the opposite VBR wall beneath the commissures (Figure 2, *L-O*).^12^^,^^13^ Intraoperative TEE was examined to assess the AR grade. Postoperative TTE was examined before discharge and followed up at outpatient clinics.

### Preoperative CT Measurements

Most CT images were acquired using contrast medium and the Aquilion ONE (Canon Medical Systems Corp) device, as previously described.^10^ The CT data for the end-diastolic phase (80% of a cardiac cycle) were imported into the image-processing system (Vitrea; Canon Medical Informatics, Inc). Manual measurements of the aortic roots and cusps were taken to assess the VBR, gHs, and eHs, as reported.^10^ The ellipticity index values were calculated using the major and minor VBR diameters as follows: ellipticity index = major diameter/minor diameter.^14^ The commissure circle area (CCA) was defined as the area of the circle on the 3 commissures (at the top of the inter-leaflet triangles). Intercommissural distances (ICDs) were measured between the commissures. The CH was the length of a perpendicular line between the VBR plane and the commissures (Figure 2).

### Automated Measurement of the Aortic Root Using CT Data

We have previously developed a deep learning−based algorithm for fully automated aortic cusp segmentation from CT images. This was achieved by combining spatial configuration-Net for detecting anatomical landmarks and U-Net for the segmentation of aortic valve components.^9^ The combined processes were trained using 258 CT volumes. We then retrained the algorithm for use with segmenting dilated roots using 67 CT volumes. There was a moderate-to-high correlation between the measurements of the algorithm and the experts, with differences of 13.4 mm^2^ for the VBR area, 2.6 mm for SOV diameter, 0.5 mm for gH, and 0.9 mm for eH.^10^ This algorithm was installed on the Vitrea workstation. Of the 40 cases in our cohort, 1 patient was excluded from the control group because of spinal metal bolts that interfered with automated root detection, and 4 were excluded because their CT data were used to train the algorithm. CT volumes were added from 15 patients who underwent VSRR after February 2016, resulting in 50 patients in the algorithm measurement group (Figure 1). The VBR, CCA, ICD, and CH were measured using the algorithm and compared with the manual measurements taken by 3 experts. This included 2 cardiac surgeons (expert 1, HY; and expert 2, MA) and 1 radiology technician (expert 3, KI) who had 9, 5, and 7 years of experience in cardiac CT data analyses, respectively. Expert 1 measured all 50 cases. Experts 2 and 3 each took measurements for 17 randomly selected cases.

### Estimation of the VBR Diameter and CP From the CT Data

Figure 2 shows the aortic root measurement parameters. The VBR diameters were calculated assuming the VBR was a circle, using the VBR area and perimeter.^4^^,^^5^^,^^15^ If the VBR exceeded 30 mm or the AR grade greater than mild, we planned to reduce the VBR diameter by 2 to 4 mm for securing sufficient cusp coaptation height (cCH),^6^ which was calculated as follows: cCH = gH – VBR diameter/2. The CPs were measured separately along the horizontal and vertical axes. Horizontally, ICDs along 3 cusps were approximately converted to cusp angles and reflected on the graft, eg, one degree-matched circumference of a 34 mm graft was 0.3 mm. Vertically, the predicted CH was approximately calculated from the measured CH, VBR, and CCA, using the Pythagorean theorem.

### Surgical Strategy and Techniques

Our modified techniques of David reimplantation was reported in detail previously^16^ and is demonstrated in Video 1. For the subvalvular sutures along the VBR line, 10 to 15 mattress 3-0 monofilament pledgeted sutures were applied in all controls and 3 patients in the CT group. In the remaining 17 CT group cases, only 6 sutures were used beneath the 3 nadirs and commissures after experiencing a case with complete atrioventricular block. We used a collagen-impregnated Dacron tube graft (Hemashield Platinum Woven Double Velour; Getinge) 30 to 34 mm in diameter. This was 6 to 8 mm larger than the planned VBR size. There are 3 reasons for our use of large-sized straight graft. These are, first, to facilitate good visualization and management of the reimplanted root structures; second, to allow free determination of the CPs in the graft; and third, to maintain vortex blood flow within the reconstructed neo-sinuses. The graft bottom was plicated to the planned VBR diameter +4 mm, considering the aortic wall width. Subvalvular VBR sutures were fixed to the graft bottom. Next, 3 commissures were temporarily attached to the graft using pledgeted sutures under the surgeon's inspection and pulled apart. This graft fabric formed ringed crimps, with the width of each one crimp being approximately 1.5 mm. This was used to estimate CHs above the VBR level. In the CT group, the CP sutures were positioned with reference to predicted CT values. In the controls, the CP sutures were flexibly aligned depending on the patients' CPs and cusp sizes, with a default height of around 25 mm and spaced horizontally at 120° intervals. Then, by the water test, if one cusp looked prolapsed, its bilateral commissures were pulled further up or apart to raise the cusp's free margin and/or the opposing CH was lowered. For remarkable cusp elongation, additional cusp repair, predominantly central plication, was applied. The graft distal portions were vertically plicated between the commissures, from the commissure level to the graft distal end, to reduce the STJ diameter matching the VBR.

### Statistical Analysis

**Figure E1:**
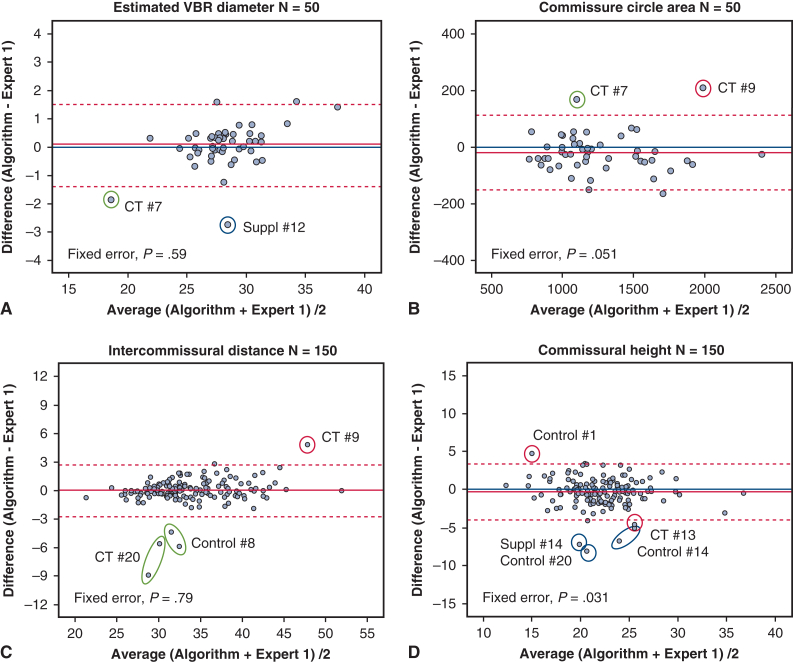
Bland-Altman plots of the manual and automatic measurement values of the patients who underwent David reimplantation. A, Estimated VBR diameter; B, commissure circle area; C, intercommissural distance; and D, commissural height. The *solid red lines* represent the means of the differences between the algorithm measurement and those of expert 1. The *dashed red lines* depict the limits of agreements (±1.96 SD). Cases that were outside the limits of agreements are marked in *blue*, *green*, or *red* on the basis of the reason for exceeding the agreement limits. *Blue* indicates poor data conditions (poor contrast or blurred images). *Green* indicates anatomical reasons such as irregular VBR shapes (CT #7), disproportionate SOVs (CT #7), and chronic type-A dissection (CT #20 and Control #8). *Red marker* indicates discrepancies between the measurement criteria of the algorithm and the expert. In the cases of CT #13 and control #1, the shadow of the cusp was fused with the thickened aortic wall on imaging, preventing accurate commissure detection. *VBR*, Virtual basal ring; *SD*, standard deviation; *CT*, computed tomography; *SOV*, sinus of Valsalva; *Suppl*, supplementary case.

**Figure E2:**
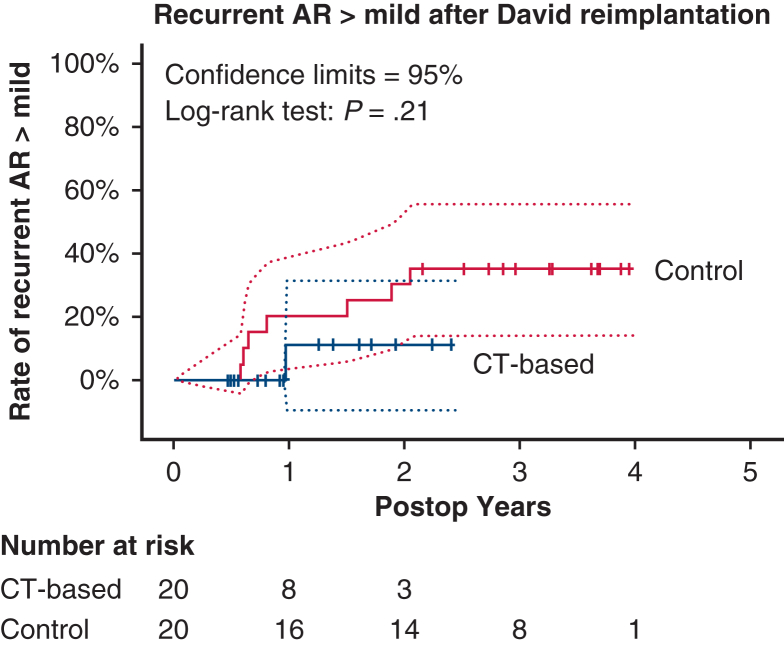
Kaplan-Meier curves for recurrent AR above the mild level after David reimplantation. The graph compares the Kaplan-Meier curve of patients whose David reimplantation planning was based on CT with that of a control group in whom conventional planning methods were used. *Dotted lines* show 95% confidence limits. The rates of recurrent AR above the mild level 1 year after David reimplantation were 11.1% in the CT group and 20.0% in the control group (Log-rank test, *P* = .21). *AR*, Aortic regurgitation; *CT*, computed tomography.

Continuous data are presented as means and standard deviations unless otherwise mentioned and were analyzed using unpaired *t* tests. Categorical data are presented as numbers and percentages and were compared using χ^2^ tests. The disproportionate ratios for the 3 measurements of the ICDs and CHs were calculated as follows: disproportionate ratio (%) = (maximal value − minimal value)/minimal value × 100. For comparisons between the algorithm's and the experts' measurements, absolute errors (AEs) and error rates were defined^10^ and shown as means and standard errors. The correlation between the 2 measurements was analyzed using the intraclass correlation coefficient (ICC) and 95% confidence interval. Bland-Altman difference plots with limits of agreement^17^ are shown in Figure E1. The fixed errors between the measurements were analyzed.^10^ Kaplan-Meier curves for recurrent AR greater than mild after David reimplantation in the CT and control groups are shown in Figure E2 and were compared using the log-rank test. SPSS for Windows, version 21 (IBM Corp) software was used for all statistical analyses.

## Results

### Demographics and Preoperative Echocardiography and CT Evaluation

Table 1 shows the demographic and echocardiographic data of the cohort. The mean age of the CT group was greater than that of the controls (*P* = .019). The majority of patients were diagnosed with connective tissue diseases (mostly Marfan syndrome). Preoperative TTE showed AR greater than mild in 20% to 30% of cases. The mean SOV diameter was larger in the CT group (*P* = .010). Table 2 shows the preoperative CT measurements. The estimated VBR diameters were similar between the groups. However, the CT group included a greater proportion of patients with an oval-shaped VBR (ellipticity index >1.2) than the controls (70% vs 45%, *P* = .11). The CT group showed a tendency to larger SOVs and ICDs, and parts of the CHs and eHs were significantly greater than those of the controls. The disproportionate ratios of the ICDs were larger in the CT than the controls (*P* = .085). The cusp angles of the LCC and RCC in the CT group were smaller (*P* = .050) and larger (*P* = .091), respectively, than those of the controls. However, the disproportionate ratios of the CHs were similar between the groups (*P* = .91).

**Table 2:** Preoperative CT measurements

Parameter	CT group (n = 20)	Control group (n = 20)	*P* value
VBR area, mm2	615.3 ± 136.7	644.5 ± 140.2	.51
VBR perimeter, mm	88.4 ± 10.0	90.4 ± 9.4	.51
Estimated VBR diameter, mm	28.0 ± 3.2	28.6 ± 3.0	.49
Ellipticity index of VBR >1.2, n (%)	14 (70%)	9 (45%)	.11
SOV diameter, mm			
Left coronary sinus	45.5 ± 5.3	43.9 ± 4.8	.32
Right coronary sinus	46.7 ± 6.3	43.5 ± 5.1	.083
Noncoronary sinus	46.2 ± 5.9	43.5 ± 4.6	.11
Disproportionate ratio (%)	7.8 ± 5.3	7.0 ± 5.9	.68
Commissure circle area, mm2	1330.7 ± 403.3	1189.2 ± 286.3	.21
ICD			
Between N-L and L-R, mm	32.0 ± 5.0	31.5 ± 4.5	.75
Between L-R and R-N, mm	36.5 ± 5.7	33.6 ± 3.5	.062
Between R-N and N-L, mm	34.8 ± 5.0	32.5 ± 4.3	.14
Disproportionate ratio, %	17.6 ± 14.7	11.3 ± 5.9	.085
Cusp angle, °			
LCC	111.5 ± 8.2	115.9 ± 5.1	.050
RCC	127.2 ± 6.2	124.2 ± 4.7	.091
NCC	121.4 ± 6.8	119.9 ± 5.1	.43
CH			
N-L, mm	22.6 ± 3.7	19.9 ± 3.0	.017∗
L-R, mm	23.0 ± 3.7	21.8 ± 3.8	.29
R-N, mm	24.9 ± 4.9	21.8 ± 4.1	.036∗
Disproportionate ratio, %	26.1 ± 20.0	25.4 ± 12.5	.91
Predicted CH, mm			
N-L	23.7 ± 3.8	20.7 ± 3.2	.012∗
L-R	24.1 ± 3.7	22.5 ± 3.7	.20
R-N	25.8 ± 5.2	22.6 ± 3.9	.032∗
Geometric height, mm			
LCC	18.6 ± 2.8	19.2 ± 2.1	.48
RCC	18.0 ± 2.0	16.9 ± 2.6	.14
NCC	20.7 ± 2.8	20.0 ± 2.9	.42
Effective height, mm			
LCC	12.9 ± 2.5	11.6 ± 1.9	.085
RCC	13.4 ± 2.6	11.5 ± 2.6	.029∗
NCC	13.3 ± 2.4	11.5 ± 2.2	.019∗

*CT*, Computed tomography; *VBR*, virtual basal ring; *SOV*, sinus of valsalva; *ICD*, intercommissural distance; *N-L*, commissures between the non and left-coronary sinuses; *L-R*, commissures between the left and right coronary sinuses; *R-N*, right and noncoronary sinuses; *LCC*, left-coronary cusp; *RCC*, right coronary cusp; *NCC*, noncoronary cusp; *CH*, commissural height.

∗ *P* < .05 was considered statistically significant.

### Operative Outcomes

**Figure 3:**
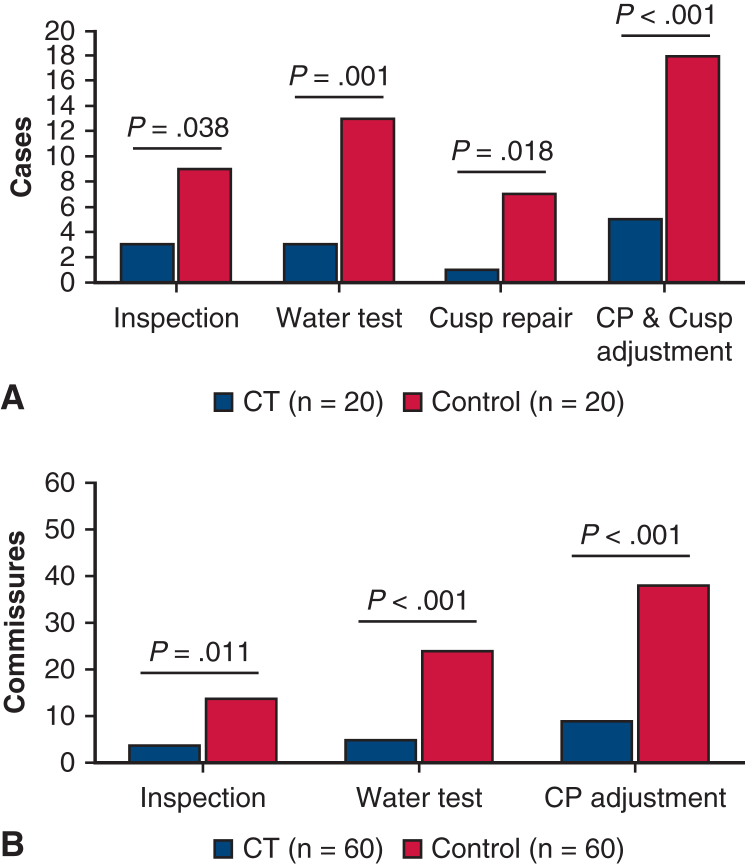
Intraoperative adjustment of the commissural positions and cusp repair during David reimplantation. The number of patients (A) and commissures (B) requiring CP adjustments based on inspection of the aortic valve and the water test, as well as those requiring cusp repair, are shown. *CP*, Commissural position.

Table 3 shows the surgical parameters. In the CT group, the aortic crossclamp time was shorter (*P* = .001), whereas the subvalvular sutures counted fewer (*P* < .001) than the controls. In the CT group, 16 patients (80%) had horizontal shift at both the commissure and VBR levels, and 19 (95%) had final uneven CHs. In the controls, horizontal CP shifting and uneven CHs occurred in 8 (40%) and 12 (60%) patients, respectively. These incidences were significantly different between the groups (horizontal CP shifting: *P* = .010; uneven CHs: *P* = .008). The cCH were similar between the groups (*P* = .36). Cusp repair was added in 1 patients in the CT group and 7 patients in the control group (*P* = .018). Figure 3 shows the number of patients and commissures requiring CP adjustments and cusp repair, which were fewer in the CT group (*P* < .001). Table E1 shows the details of the 5 cases in the CT group necessitating CP adjustments. CHs were adjusted by inspection and water test in 3 and 2 cases, respectively. The case CT #2 also needed cusp repair. In the whole CT group, 6 of the 20 patients had an average SOV >50 mm, and 4 of these required CP adjustments. Of the 14 patients with SOV <50 mm, only 1 patient required CP adjustment (*P* = .005). There was no case with AR greater than mild in either group after weaning off cardiopulmonary bypass.

**Table 3:** Surgical parameters of David reimplantation

Parameter	CT group (n = 20)	Control group (n = 20)	*P* value
Operation time, min	397.9 ± 88.0	439.3 ± 72.9	.11
Cardiopulmonary bypass time, min	224.5 ± 45.1	261.7 ± 47.4	.015∗
Aortic crossclamp time, min	173.8 ± 26.9	209.1 ± 37.0	.001∗
Resternotomy, n (%)	1 (5%)	2 (10%)	.55
Concomitant procedure, n (%)	8 (40%)	3 (15%)	.077
Coronary artery bypass grafting	1	0	
Pulmonary valve isolation	2	0	
Partial arch replacement	1	0	
Thoracoplasty for funnel chest	4	3	
Graft size, mm			.41
30	1	2	
32	4	7	
34	15	11	
Graft bottom size, mm	30.6 ± 2.2	29.3 ± 2.0	.056
Subvalvular sutures	7.0 ± 2.6	13.1 ± 1.3	<.001∗
Horizontal shift of CP			
VBR level, n (%)	16 (80%)	0	<.001∗
Commissure level, n (%)	16 (80%)	8 (40%)	.010∗
Final CHs (mm) including 2 mm widths of pledgets			
N-L commissure	25.6 ± 3.6	25.0 ± 1.7	.46
L-R commissure	26.2 ± 3.8	24.9 ± 2.2	.20
R-N commissure	27.6 ± 4.1	25.6 ± 1.9	.052
Final uneven CH, n (%)	19 (95%)	12 (60%)	.008∗
Cusp coaptation height, mm	5.8 ± 1.9	5.2 ± 2.2	.36
Additional cusp repair, n (%)	1 (5%)	7 (35%)	.018∗
Central plication	1	7	
Subcommissural plication	0	1	
STJ plication, mm	29.0 ± 2.3	26.6 ± 0.9	<.001∗

*CT*, Computed tomography; *CP*, commissural position; *VBR*, virtual basal ring; *CH*, commissural height; *N-L*, commissures between the non and left-coronary sinuses; *L-R*, commissures between the left and right coronary sinuses; *R-N*, right and noncoronary sinuses; *STJ*, sinotubular junction.

∗ *P* < .05 was considered statistically significant.

### Postoperative Course

There were no hospital deaths or major postoperative adverse events in either group. Befire discharge, none of the patients had AR graded as greater than mild by TTE. Figure E2 presents the Kaplan**-**Meier curves for cases of recurrent AR greater than mild, accounting for 11.1% of the CT group and 20.0% of the control group after 1 year (*P* = .21).

### Automated Measurement of the VBR Dimensions and CPs

**Figure E3:**
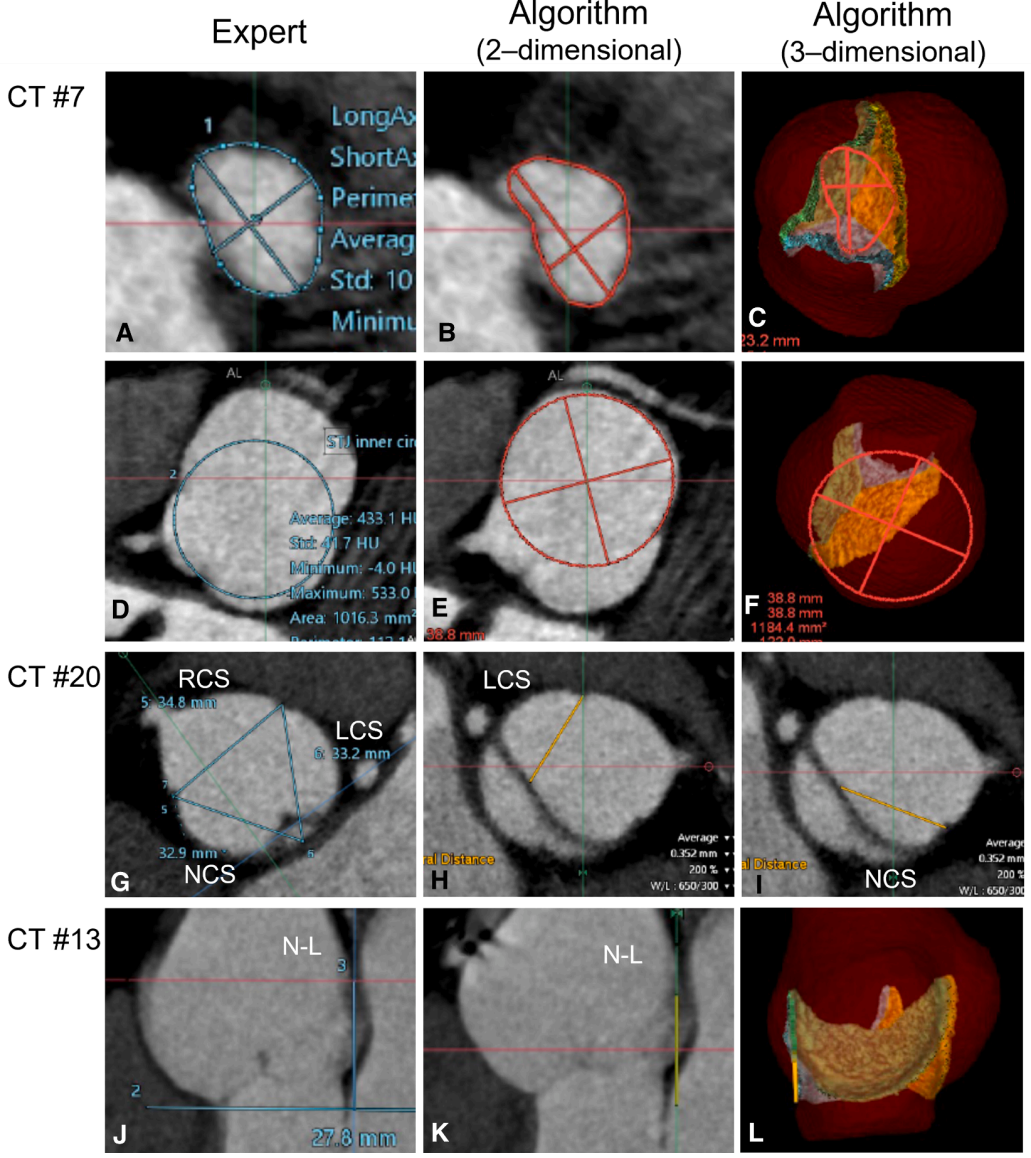
Representative cases of outliers from the Bland-Altman plots of the measurement values of patients who underwent David reimplantation. A-C, The algorithm's automated measurements underestimated irregular VBR in case CT #7. D–F, In the same patient (CT #7), the algorithm wrongly detecting the commissure positions in disproportionately dilated SOVs. G-I, In a patient with chronic type A dissection (CT #20), the algorithm identified the commissural positions for the ICD measurement on the intimal flap, whereas expert 1 set them at their original position, assuming that the aortic wall was not dissected. CT #7 and CT #20 are marked in *green* on the Bland-Altman plots (Figure E1), indicating anatomical reasons for the measurement discrepancies. J-L, In CT #13 the cusps' shadow was fused with the thickened aortic wall around the commissure on imaging, preventing the accurate detection of commissures. This case is marked in *red* on the Bland-Altman plots, indicating differences in the measurements criteria of the algorithm and the expert. The manual measurements (A,D, G, J), automated measurements from 2-dimensional images (B, E, H, K), and automated measurements from 3-dimensional images (C, F, I, L) were compared. *VBR*, Virtual basal ring; *CT*, computed tomography; *SOV*, sinus of Valsalva; *ICD*, intercommissural distance; *RCS*, right-coronary sinus; *LCS*, left-coronary sinus; *NCS*, noncoronary sinus; *N-L*, commissures between the non and left coronary sinuses.

Table 4 compares the measurements of our algorithm and expert 1. The AE (error rate) of the estimated VBR diameters, ICDs, and CHs were 0.5 to 1.3 mm (1.7%-6.2%), corresponding to an almost-perfect correlation (ICC of 0.87-0.97). In our Bland-Altman plots (Figure E1), the patients with values that fell outside the limits of agreements could be explained by poor data conditions, anatomical reasons, and discrepancies between the measurement criteria of the algorithm and the experts. Representative cases of such outliers are shown in Figure E3. Table E2 provides a comparison of the CT measurements by the 3 experts. The AEs between expert 1 and the algorithm were not more than those between the experts in any parameter. The ICCs between the experts were excellent, except for CHs (0.77-0.83). Even for these, the average values of the experts and those of the algorithm showed an excellent ICC (0.91).

**Table 4:** Interobserver variability between the CT measurements by expert 1 and a deep learning−based algorithm

Parameter	Case n	Expert 1 [mm^2^ or mm]	Algorithm [mm^2^ or mm]	Difference [mm^2^ or mm]	AE (SE) [mm^2^ or mm]	ER (SE) [%]	ICC (95% CI)
VBR area	50	621.3 ± 124.8	616.5 ± 139.2	−4.9 ± 36.3	22.8 (4.0)	3.8 (0.7)	0.96 (0.94-0.98)
VBR perimeter	50	88.9 ± 8.9	89.7 ± 9.8	0.8 ± 2.1	1.7 (0.2)	1.9 (0.2)	0.97 (0.94-0.98)
Estimated VBR diameter	50	28.2 ± 2.8	28.2 ± 3.1	0.1 ± 0.7	0.5 (0.1)	1.7 (0.3)	0.97 (0.95-0.98)
Commissure circle area	50	1271.5 ± 354.7	1252.3 ± 353.6	−19.2 ± 67.9	53.4 (6.4)	4.3 (0.5)	0.98 (0.97-0.99)
Intercommissural distance	150	33.7 ± 4.9	33.7 ± 5.2	0.0 ± 1.4	0.8 (0.1)	2.5 (0.3)	0.96 (0.95-0.97)
Commissural height	150	22.4 ± 3.8	22.0 ± 3.6	−0.3 ± 1.9	1.3 (0.1)	6.2 (0.5)	0.87 (0.82-0.90)

*AE*, Absolute error; *SE*, standard error; *ER*, error rate; *ICC*, intraclass correlation coefficient; *CI*, confidence interval; *VBR*, virtual basal ring.

## Discussion

High-resolution CT is a valuable tool for analyzing aortic root structures.^3^, ^4^, ^5^, ^6^ VBR areas and perimeters are regularly used in transcatheter aortic valve replacements for valve selection. Although such CT-based VBR measurements have been proposed for VSRR graft sizing,^4^^,^^10^ to our knowledge, no report has yet clinically applied this method. As the controls, TEE determined the VBR diameters as it provides better estimates of the oval-shaped VBR than 2-dimensional TTE.^12^^,^^13^ In comparing the CT and TEE estimates of VBR diameters (n = 34), we found that CT produced slightly larger estimates, with a difference of 0.1 ± 2.3 mm and ICC of 0.79 (0.61-0.89), suggesting a substantial correlation.

The STJ dimensions were reduced in VSRR, whereas our approach is to preserve the interrelationships between the 3 disproportionate CPs.^3^^,^^4^^,^^12^ Even with discrepant cusp angles, the CT group required less CP adjustment than the controls. Although low eHs could have affected surgeons’ intraoperative decisions with the patients in the control group (Table 2), both groups showed similar cCHs (5-6 mm) (Table 3). Although our target cCH might be insufficient on the basis of the recent recommendation of 8 to 9 mm,^18^ the CT group showed promising midterm outcomes.

This CT-based approach can simplify surgery and shorten aortic crossclamp time, but the lower number of subvalvular sutures might affect the time (Table 3). The aortic crossclamp times of the CT group patients with 6 subvalvular sutures (n = 17) were an average of 10 minutes shorter than those of the patients with >10 sutures (n = 3) (172 ± 29 minutes vs 182 ± 9 minutes). This difference roughly matched the time for the rest of the 4 to 9 subvalvular sutures. The average time difference for the aortic crossclamping between the control and CT groups was much greater (35 min), presumably owing to the CT-based planning in the CT group.

Even in the CT group, 5 cases needed CP adjustments. Large SOVs (>50 mm) leaded to decreased accuracy in predicting CPs, because CPs were defined by assuming the round VBR shape (Figure 2, *C*). The gaps errors between the oval-shaped VBR and CCA would be evident with SOV >50 mm. For more precise CP predictions, the cusp angles and the predicted f-value for the CHs necessitates direct measurement using complicated manual methods.

Deep learning−based automated measurement of aortic roots has been progressing^9^^,^^10^^,^^12^^,^^19^ and focusing on transcatheter aortic valve replacement planning.^19^ To our knowledge, we first assessed the feasibility of a fully automated algorithm for measuring CCAs, ICDs, and CHs and found excellent correlations between the algorithm and expert measurements, even better than interobserver variance of experts (Table 4 and Table E2). The presence of AR did not affect the algorithm's performance.

VSRR is a complex procedure demanding an interplay between techniques and art. Its proper execution requires both fundamental surgical training and a great deal of experience. The CT-based logical approach we delineate can be used for an invaluable training tool, as well as decision-making by a surgical team, for successful VSRR.

The present study had some limitations. First, this was a retrospective single-center study so the effects of the learning curve for the procedure could not be completely excluded. Second, the sample size was small. Although this was a proof-of-concept study that aimed to demonstrate the applicability of a CT-based approach to VSRR, further studies with larger samples and more variations are needed to refine our strategy and algorithm. Third, since bicuspid valves configure 2 CPs with or without a raphe, the angles of the respective cusps are necessary to determine the horizontal CPs. Fourth, we excluded cases in which aortic cusp repair for cusp deformity was planned. Finally, the long-term outcomes for the spared valve function needs further investigation.

## Conclusions

Our results show that CT-based VSRR planning can assist in the intraoperative determination of graft sizes and CPs and reduce the need for additional cusp repair. This simplification of the intraoperative process can reduce the cardiac arrest time. Our artificial intelligence-based algorithm can replace expert measurements to support standardized VSRR planning.

## Conflict of Interest Statement

H. Yamauchi, I. Sakuma, and M. Ono received research grants from Canon Medical Systems Corporation. H. Yamauchi received the image processing system (Vitrea) for research purposes from Canon Medical Informatics, Inc. G. Aoyama is Canon Medical Systems Corporation employee. All other authors reported no conflicts of interest.

The *Journal* policy requires editors and reviewers to disclose conflicts of interest and to decline handling or reviewing manuscripts for which they may have a conflict of interest. The editors and reviewers of this article have no conflicts of interest.

## Figures and Tables

**Table E1:** Cases of commissural position adjustment in the CT group

Case#	SOV diameter. mm left, right, non	Preoperative AR grade	Graft size, mm	CH by inspection, mm			Cusp condition after inspection	CP changed by water test	Additional cusp repair
				N-L	L-R	R-N			
CT #2	51, 54, 50	Mild	32^H^	28	27	39 to 36^V^	RCC prolapse	H: L-R to LCC (2 mm)	RCC central plication
								H: R-N to NCC (2 mm)	
CT #4	52, 55, 61	Mild	34	35 to 30^V^	33	41 to 37^V^	even	no	No
CT #6	54, 52, 52	Mild	34	28	28	22	NCC prolapse	V: L-R (28 to 27 mm)	No
CT #9	57, 65, 58	Severe	32^H^	28	28	30	LCC prolapse	V: R-N (30 to 28 mm)	No
								H: L-R to RCC (2 mm)	
CT #16	46, 45, 47	Trivial	34^H^	28	28 to 25^V^	28	Even	No	No

Intraoperative CH includes 2 mm width of pledgets. *SOV*, Sinus of valsalva; *AR*, aortic regurgitation; *CH*, commissural height; *N-L*, commissures between the non and left-coronary sinuses; *L-R*, commissures between the left and right coronary sinuses; *R-N*, right and noncoronary sinuses; *CP*, commissural position; *RCC*, right coronary cusp; *LCC*, left-coronary cusp*;NCC*, noncoronary cusp. ^H^Horizontal shift; ^V^Vertical shift.

**Table E2:** Correlation of CT measurement values among 3 experts and the algorithm

Parameter	Comparison	Difference [mm^2^ or mm]	AE (SE) [mm^2^ or mm]	ER (SE) [%]	ICC (95% CI)
VBR area	Expert 1 vs algorithm	−12.9 ± 14.7	15.7 (2.8)	2.5 (0.4)	0.97 (0.83-0.99)
(n = 17)	vs expert 2	−11.0 ± 16.3	14.7 (3.1)	2.6 (0.6)	0.98 (0.91-0.99)
	vs expert 3	−1.0 ± 17.3	13.0 (2.7)	2.3 (0.5)	0.98 (0.95-0.99)
	Expert (mean) vs algorithm	−8.9 ± 18.1	16.9 (2.5)	2.8 (0.4)	0.97 (0.92-0.99)
VBR perimeter	Expert 1 vs algorithm	0.6 ± 1.0	0.9 (0.2)	1.1 (0.2)	0.98 (0.93-0.99)
(n = 17)	vs expert 2	−0.4 ± 1.6	1.2 (0.2)	1.4 (0.3)	0.97 (0.92-0.99)
	vs expert 3	0.5 ± 1.6	1.1 (0.3)	1.3 (0.3)	0.97 (0.91-0.99)
	Expert (mean) vs algorithm	0.6 ± 1.3	1.1 (0.2)	1.2 (0.3)	0.97 (0.93-0.99)
Estimated VBR diameter	Expert 1 vs algorithm	0.0 ± 0.3	0.3 (0.0)	0.9 (0.2)	0.99 (0.96-1.00)
	vs expert 2	−0.2 ± 0.4	0.4 (0.1)	1.3 (0.3)	0.97 (0.92-0.99)
(n = 17)	vs expert 3	0.1 ± 0.4	0.3 (0.1)	1.0 (0.2)	0.98 (0.93-0.99)
	Expert (mean) vs algorithm	0.0 ± 0.4	0.3 (0.1)	1.2 (0.2)	0.98 (0.94-0.99)
Commissure circle area	Expert 1 vs algorithm	−9.2 ± 73.1	52.1 (12.2)	4.2 (0.8)	0.99 (0.97-1.00)
	vs expert 2	77.7 ± 112.1	87.9 (25.2)	6.8 (1.7)	0.96 (0.83-0.99)
(n = 17)	vs expert 3	42.1 ± 92.5	79.5 (14.8)	6.2 (1.1)	0.98 (0.93-0.99)
	Expert (mean) vs algorithm	−49.1 ± 62.4	60.5 (12.3)	4.9 (1.0)	0.99 (0.92-1.00)
Intercommissural distance	Expert 1 vs algorithm	0.2 ± 1.1	0.8 (0.1)	2.2 (0.3)	0.98 (0.97-0.99)
	vs expert 2	0.3 ± 1.0	0.8 (0.1)	2.3 (0.3)	0.99 (0.97-0.99)
(n = 51)	vs expert 3	1.2 ± 1.1	1.4 (0.1)	4.2 (0.3)	0.96 (0.59-0.99)
	Expert (mean) vs algorithm	−0.3 ± 1.1	0.9 (0.1)	2.7 (0.3)	0.98 (0.97-0.99)
Commissural height	Expert 1 vs algorithm	0.1 ± 1.5	1.2 (0.1)	5.7 (0.8)	0.91 (0.85-0.95)
(n = 51)	vs expert 2	−0.2 ± 2.5	1.9 (0.2)	8.6 (1.2)	0.83 (0.72-0.90)
	vs expert 3	−1.0 ± 2.5	2.1 (0.2)	10.5 (1.3)	0.77 (0.60-0.87)
	Expert (mean) vs algorithm	0.6 ± 1.4	1.2 (0.1)	5.8 (0.7)	0.91 (0.83-0.95)

*AE*, Absolute error; *SE*, standard error; *ER*, error rate; *ICC*, intraclass correlation coefficient; *CI*, confidence interval; *VBR*, virtual basal ring.

## Supplementary Data

Video 1 CT measurements and the David reimplantation procedure. Video available at: https://www.jtcvs.org/article/S2666-2507(25)00544-9/fulltext.
