# Supplementary figures and images for: Computed tomography−based prediction of commissural positions facilitates valve-sparing aortic root replacement
Source: JTCVS Tech. 2025 Nov 27;35:102163. doi: 10.1016/j.xjtc.2025.11.007 (PMC12881777; doi:10.1016/j.xjtc.2025.11.007)

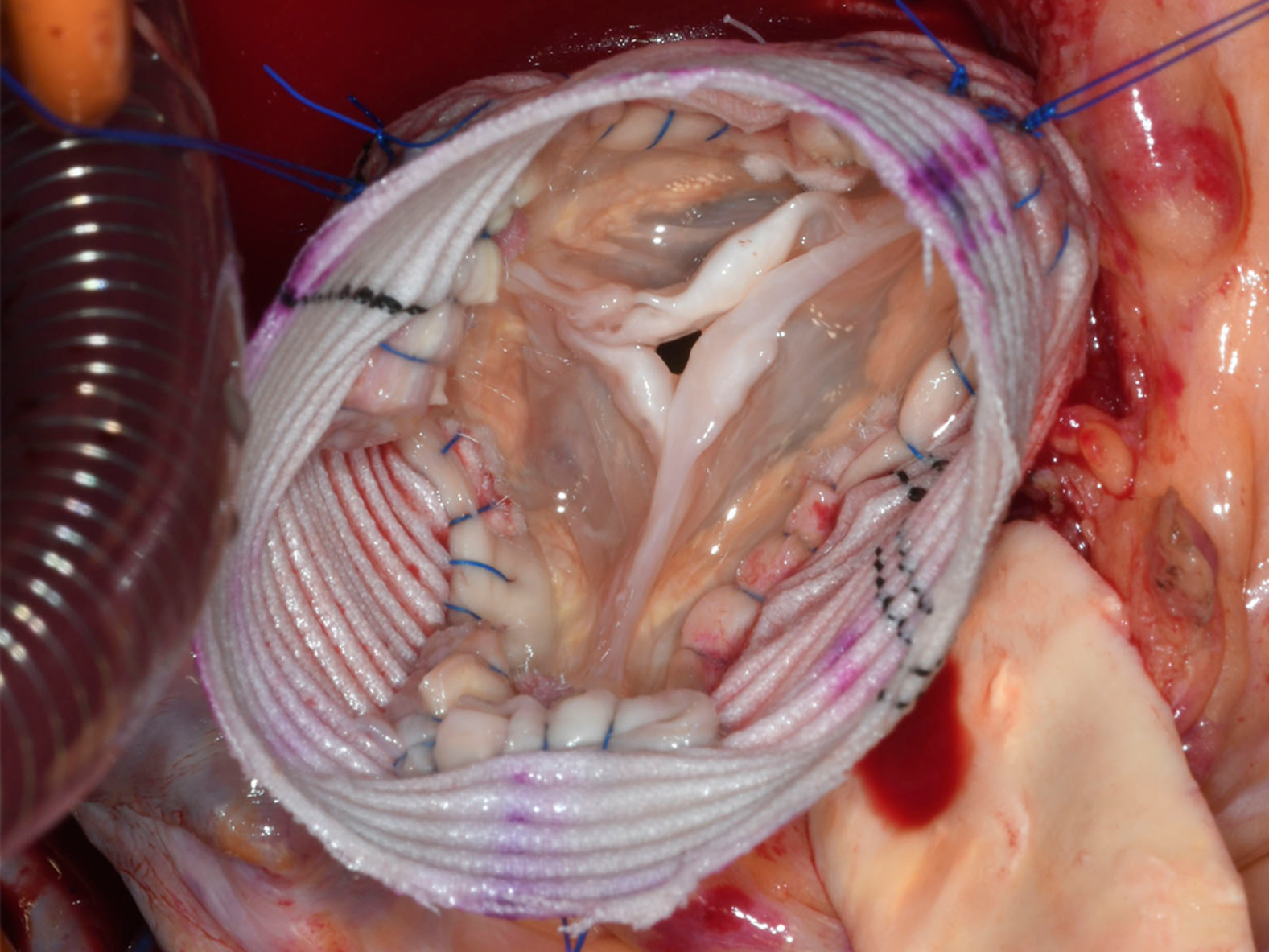

Supplement: Video 1 — CT measurements and the David reimplantation procedure. Video available at: https://www.jtcvs.org/article/S2666-2507(25)00544-9/fulltext. [file fx2.jpg]
